# Assessing service usage and protective factors in a pediatric psychiatry clinic during the COVID-19 pandemic

**DOI:** 10.3389/fpsyg.2024.1354544

**Published:** 2024-07-29

**Authors:** A. J. Caruso, A. Basu, T. H. Urban, M. M. Kaskas, N. Rotter, J. Wozniak, D. Friedman

**Affiliations:** ^1^Department of Psychiatry, Massachusetts General Hospital/Harvard Medical School, Boston, MA, United States; ^2^Department of Clinical Psychology, Graduate School of Medical Sciences, Tottori University, Tottori, Japan

**Keywords:** protective factor, resilience, child and adolescent mental health, pediatric psychiatry, service usage, COVID-19 pandemic

## Abstract

Youth with developmental and pre-existing mental health conditions have been particularly vulnerable to declines in psychological functioning during the COVID-19 pandemic. This study aimed to first, analyze service usage within an outpatient child and adolescent psychiatry clinic in the months preceding and during the COVID-19 pandemic, and second, to examine associations with potential protective factors against mental health concerns in a treatment-engaged sample. Service usage was examined using clinic billing data, and reports on protective factors were gathered via parent survey of 81 children ages 6–17 years who received mental health treatment in an outpatient psychiatry clinic during the pandemic. Protective factors were assessed at the individual, family, and community levels, and included children’s use of coping strategies, parental resilience, and parents’ perceived social supports. Study outcomes, including mental health concerns, mental health emergencies, pandemic-related distress, and social impact of the pandemic, were analyzed via Pearson correlations and simultaneous multiple linear regressions. Findings suggest increased service usage and child coping, parental resilience, and social connectedness as factors associated with fewer mental health concerns in youth with psychiatric concerns during the pandemic. This study lends support for expanding psychiatric services with continued use of telemedicine platforms. Further, findings suggest a mental health benefit to optimizing individual, parental, and community-based resources to enhance children’s psychological functioning, particularly for youth with pre-existing mental health conditions.

## 1 Introduction

The impact of the coronavirus disease 2019, known as the COVID-19 pandemic, on child and adolescent mental health, healthcare systems, and psychiatric treatment remain important areas of investigation. The first cases of COVID-19 in the United States were confirmed in January 2020, but cases accelerated quickly as of February–March 2020, unleashing a series of public health responses including travel restrictions, quarantine measures, screenings, public health risk assessments, and more (Schuchat, 2020). General population studies suggest increases in youth depressive and anxious symptoms compared to pre-pandemic estimates (Kauhanen et al., 2022; Samji et al., 2022). In particular, children and adolescents with developmental and pre-existing mental health conditions have experienced more significant declines in psychological functioning compared to those without (Neelam et al., 2021; Theberath et al., 2022). Additionally, an increase in the number of psychiatric visits across both ambulatory and emergency department (ED) settings, along with increased severity of psychiatric illness, have corresponded to more frequent medical admissions and extended inpatient psychiatric unit stays (Leith et al., 2022; Shankar et al., 2022).

Increased access to child and adolescent psychiatric services was made possible in the US in large part due to the expansion of telemedicine under emergency orders (Barney et al., 2020; Centers for Medicare and Medicaid Services, 2021; Folk et al., 2022). However, demand continues to outpace availability (Shidhaye, 2023). The COVID-19 pandemic intensified the need for psychiatric services and exploration of avenues available to increase access to care (Benton et al., 2021). Yet, there remain gaps in our understanding of telehealth utilization for pediatric psychiatric services from pre-to mid-pandemic to the reopening of in-person care. Assessing trends in telehealth usage may inform future planning around mental health service delivery needs, and thus, was a goal in the present study. Alongside, pandemic-related stress and social isolation have been associated with worsening psychiatric symptomatology in youth (Deolmi and Pisani, 2020). Attention has been drawn to promoting *resilience*, which may be viewed both as an individual’s ability to adapt in the face of hardship, as well as a more systems-level process to evolve amidst multisystem challenges (Masten et al., 2021). An expanding literature highlights factors that may protect against mental health decline and facilitate resilience, including children’s utilization of more *active* coping strategies—whereby “purposeful, volitional efforts” are taken to regulate oneself when met with adversity (e.g., engaging in physical activity, spending time outdoors, adhering to daily routines; Compas et al., 2012, p. 458)—and parents’ own perceptions of their resilience and support from social networks (Shoshani and Kor, 2022; Urban et al., 2022). Thus, the identification and optimization of protective factors that are known to enhance children’s mental health, reduce pandemic-related distress, and increase social adjustment, especially for those more at-risk for deteriorations in functioning, remain key areas of investigation.

This study had two objectives. First, we sought to expand the research examining trends in psychiatric service usage in an outpatient child and adolescent psychiatric clinic over an almost three-year period (pre-pandemic through September 2022). We hypothesized that service usage in pediatric psychiatry would increase throughout the duration of the pandemic. Second, we evaluated three potential protective factors—child coping, parental resilience, and parents’ perceived social supports—in a sample of youth engaged in outpatient psychiatric treatment. For this aim, we hypothesized that greater use of active coping tools by children, parent report of their own resilience, and parental perceptions of social supports, would be associated with lower mental health-related sequalae in treatment-engaged youth. Our overarching goal was to identify individual (child) and parent-level factors that might protect against pandemic-related sequelae within a clinical mental health population.

## 2 Materials and methods

### 2.1 Participants

Data come from an outpatient child and adolescent psychiatric clinic in a large, urban academic hospital in the northeast United States. This clinic offers evidence-based psychotherapies (e.g., Cognitive-Behavioral Therapy, Dialectical-Behavioral Therapy, Acceptance and Commitment Therapy, Exposure and Response Prevention, behavioral medicine, among others) and psychopharmacological treatments (e.g., antidepressant, antipsychotic, and stimulant medications) to treat a wide range of pediatric mental health conditions. Service use data (i.e., number of monthly clinic visits) were collected from October 2019 through September 2022 by retrieving outpatient billing data. To evaluate family’s experiences during COVID-19, a total of 1,220 families of which the child or adolescent was receiving psychotherapy and/or psychopharmacology services in the psychiatry department were sent electronic messages through their patient portal with a link to the questionnaire. Eighty-one parents/guardians of children, ages 6 to 17 years old (*M* = 13.4, SD = 3.1), completed surveys regarding the experience of the pandemic between March 2021 through June 2021.

Of those who completed the survey (*N* = 81), the average child age was 13.4 years (*SD* = 3.1). 38.3% of children in the sample were between the ages of 6–11 years, while 61.7% were in the 12–17-year age range. Most patients in the sample identified as female at birth (56.8%), cisgender (93.8%), and non-Hispanic White (65.4%); 6.2% were gender expansive (i.e., gender identity listed as transgender, non-binary, genderqueer, or other gender diverse), 13.6% identified as Non-Hispanic Biracial or Multiracial, and 12.4% identified as Hispanic. More than half of the sample (64.2%) was diagnosed with an anxiety disorder, 44.0% had a diagnosis of Attention-Deficit/Hyperactivity Disorder (ADHD), and 43.2% had a diagnosed mood disorder. Other diagnoses (71.6%) include eating disorders, Posttraumatic Stress Disorder (PTSD), elimination disorders, and gender dysphoria, among others. Most children had more than one mental health diagnosis (66.7%). Parent participants in the study were mostly mothers (90.1%). The majority of parents had private insurances (84.0%), yet over a third of the sample (35.8%) reported the family’s finances were negatively impacted due to the pandemic (e.g., loss of job or savings) and 13.6% of families were concerned about ability to afford essential expenses (such as medicine). 17.3% of families reported food insecurity as well as concerns about ability to pay rent/mortgage. For full descriptive information of the study sample, please refer to the Urban et al. (2022) study.

### 2.2 Recruitment and procedure

This study was approved by the hospital’s Institutional Review Board (Protocol # 2020P004065) and carried out in accordance with the latest version of the Declaration of Helsinki. Parents were invited to participate in an online, cross-sectional survey about their own and their children’s pandemic-related experiences via a secured hospital-based electronic communication system. Parents who were not enrolled in this portal were informed of the study by phone or during clinical visits by the psychologist or psychiatrist. Inclusion criteria were (a) parents of children, ages 0 to 17 years receiving mental health services in the outpatient clinic between March 2019 (i.e., within one year prior to the pandemic) and February 2021, (b) parents could complete surveys in English and (c) parents had consented to being contacted for research studies, per hospital policy. Interested participants provided informed consent following explanation of study procedures. Parents were then sent study questionnaires electronically and entered their responses directly into a web-based database, REDCap (Research Electronic Data Capture)—a secure, Health Insurance Portability and Accountability Act (HIPAA)-compliant, electronic application. Participant surveys were identified using numbers (not names) and were stored separately from forms with identifiable information. Electronic data were stored on password-protected and encrypted computers issued through the hospital. Only members of the research team were able to access participant questionnaires. Upon completion of the study, participants were entered into a raffle to earn a gift card.

### 2.3 Measures

#### 2.3.1 Demographic information

Parents reported sociodemographic information including child age, race/ethnicity, sex assigned at birth, gender identity, parent gender identity and whether their family was experiencing financial concerns. Health insurance type (public versus private) and the child’s most recent mental health diagnoses (based on clinician documentation) were obtained from electronic medical record review.

#### 2.3.2 Child coping

Child coping was assessed by a survey created for this study (see also Urban et al., 2022). Parents reported on how often their children utilized a list of 17 psychological and behavioral coping strategies to cope with pandemic-related stress, including staying physically active, focusing on hobbies, and connecting with mental health professionals, rating their use of strategies on a 3-point Likert scale (“not at all” to “a great deal”). A total sum score, ranging from 0 to 68, was created for each participant, with higher scores indicating greater use of coping strategies. Internal consistency of this measure was good (α = 0.75).

#### 2.3.3 Parental resilience

Parents completed the 10-item Connor-Davidson’s Resilience Scale (CD-RISC-10; Connor and Davidson, 2003). Items included, “I am able to adapt to change” and “I am not easily discouraged by failure.” Items were rated on a 5-point Likert scale from “not very true” to “true nearly all the time.” Standard scoring was employed to create a sum score ranging from 0 to 40 for each participant. The CD-RISC-10 has good internal consistency and construct validity, including excellent scale reliability in the current study (α = 0.92).

#### 2.3.4 Parental perceptions of social support

Parents rated their perceptions of social support during the pandemic using a survey adapted from the validated Duke Social Support and Stress Scale (Parkerson et al., 1991). Parents responded on a 5-point Likert scale from “not at all” to “extremely” regarding how supported they felt by their spouse/partner/significant other, child(ren), parents, extended family, friends, neighbors, co-workers, or another source of support. A mean score of all items represented perceptions of overall social support during the pandemic, with higher scores indicating greater social supports. Internal consistency for this measure was strong (α = 0.89).

#### 2.3.5 Child mental health concerns and emergencies

Parents were asked whether their children’s mental health concerns got better (1), stayed the same (2), or got worse (3) during the pandemic. Parents also responded “yes” or “no” to whether their child had any mental health emergencies during the pandemic. If applicable, parents rated which type of help they sought, such as in-person emergency services (e.g., inpatient/overnight mental health program, day program), virtual emergency mental health services (e.g., virtual day program), other sources of help, or whether they were unable to get the help they needed or if help was delayed.

#### 2.3.6 Pandemic-related distress

Pandemic-related distress was a single item measure of parents’ perceptions of their child’s level of distress caused by the COVID-19 pandemic on a 0–100 scale, akin to the commonly used subjective units of distress scale (SUDS; e.g., Demetillo et al., 2021). Higher scores indicated higher perceived distress.

#### 2.3.7 Social impact

Parents were asked to rate the extent to which their child was socially impacted by the pandemic on a 7-item measure developed for this study. On a 5-point Likert scale from not at all (0) to a great deal (4), parents reported on children’s missed activities, events, and personal connection (e.g., graduations, travel, religious services) due to the pandemic. Parents also rated how much their child missed going to school, work, or seeing friends or family in-person. A mean score of items was calculated, with higher scores indicating a more negative social impact of the pandemic. Internal consistency for this measure was strong (α = 0.85).

### 2.4 Statistical approach

To determine sample size, we conducted power analyses using G*Power 3.1.9.2 (Faul et al., 2009). Power analyses indicated that a sample size of at least 74 participants was required to achieve a medium effect size (f^2^ = 0.15) in a model with two predictor variables (the independent variable and child age, the covariate in study), assuming alpha = 0.05 and power = 0.95. Thus, our sample size of 81 was sufficiently powered to conduct the analyses performed in the study.

Statistical analyses were conducted in SPSS (IBM: Version 28). First, we examined means and standard deviations of study variables (protective factors, child mental health concerns and emergencies, distress, and social impact of pandemic). Next, Pearson correlations were computed to assess whether demographic variables (child age, gender, ethnicity, insurance type as proxy for family income) related to the study variables to determine whether to include these variables as covariates in primary analyses. Only *child age* was significantly related to outcome variables and was therefore retained in analyses. Finally, simultaneous multiple linear regressions were conducted with study outcomes (mental health concerns, mental health emergencies, pandemic-related distress, and social impact of the pandemic) as the dependent variables. The significance threshold for analyses was *p* = 0.05.

## 3 Results

### 3.1 Outpatient psychiatry service use data, October 2019 – September 2022

**Figure 1:**
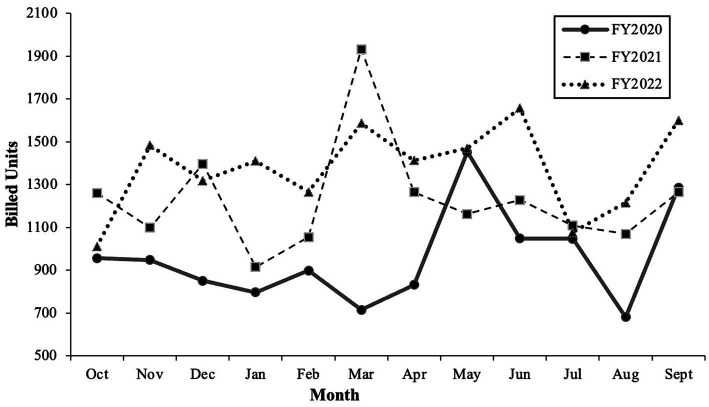
Service use data in outpatient psychiatry department. Fiscal year (FY) spans from October of the previous year to September of the named year. For example, FY2020 consists of October 2019 through September 2020. Of note, the first COVID-19 cases in the United States were confirmed in January 2020, and the number of cases accelerated as of February–March 2020 (Schuchat, 2020). By September 2022, COVID-19 vaccines, treatments, prevention tools, and infection-induced immunity reduced the risk for serious COVID-19 illness and allowed for safer returns to in-person activities, including medical visits and schooling (Massetti et al., 2022).

Figure 1 summarizes the number of completed monthly and annual clinic visits in our Child and Adolescent Outpatient Psychiatry Department (OPD) over an almost three-year period (Fiscal Years 2020–2022), including the months before the COVID-19 pandemic. The number of annual visits completed by psychiatrists, psychologists, and psychiatry and psychology trainees in the OPD increased by 28.0% between Fiscal Year 2020 and 2021 and again by 11.8% between Fiscal Year 2021 and 2022. Regarding telehealth usage, only 7% of clinic visits were conducted using remote platforms between October 2019 and February 2020. By March 17, 2020, 100% of visits were telehealth. In September 2022, 48.2% of visits were telehealth, and the remaining 51.8% of visits were in-person.

### 3.2 Impact of the pandemic and emergency service use

When asked how children’s mental health concerns changed during the pandemic, 23.3% of parents reported their children’s mental health “got better,” 24.7% reported “stayed the same,” and 52.1% reported their children’s mental health “got worse.” Additionally, 23.0% of parents reported that their child had a mental health emergency. Of these children who had a mental health emergency, 41.2% of their parents reported using *in-person* emergency mental health services, 17.6% reported using *virtual* emergency mental health services, and 41.2% indicated using “other sources of help.”

Regarding negative social impact, parents rated above the midpoint of the scale for each item, indicating at least “somewhat” of a negative social impact. The highest rated items included “missing seeing family or friends due to ‘social distancing’ or travel restrictions” (*M =* 3.54; *SD* = 0.92) and “missing important events or experiences” (*M =* 3.10; SD = 1.14). Across all domains, parents rated “much” of a negative social impact (*M* = 2.97, SD = 0.92) overall.

### 3.3 Preliminary analyses

Next, we examined correlations between demographic and study variables (Table 1). Given the large age range in the sample, child age was defined *a priori* as a covariate; the goal was to evaluate relations among variables above and beyond any variance attributed to child age. In correlation analyses, child age was significantly related to children’s use of coping strategies (*r = 0*.23, *p* < 0.05); 95% CI [0.01, 0.43], such that older children were more likely to use coping strategies during the pandemic (see Table 1).

**Table 1:** Means, standard deviations, and correlations among study variables.

	*M*	(SD)	1	2	3	4	5	6	7	8
1. Child age	13.4	(3.10)	1.00	0.23*	0.14	0.01	−0.10	0.14	0.15	−0.16
2. Child coping	1.37	(0.52)		1.00	0.09	0.22+	−0.12	−0.06	−0.17	−0.21+
3. Parent resilience	2.85	(0.63)			1.00	0.39***	−0.28*	−0.16	−0.18	0.10
4. Social support	2.09	(0.97)				1.00	−0.30*	−0.25*	−0.25*	0.14
5. MH concerns	2.29	(0.83)					1.00	0.24*	0.42***	−0.02
6. MH emergencies	0.23	(0.42)						1.00	0.49***	−0.10
7. Pandemic distress	60.5	(23.0)							1.00	0.31*
8. Negative social impact	2.97	(0.92)								1.00

*N* = 81. + *p* < 0.10. *p < 0.05. **p < 0.01. ***p < 0.001.

### 3.4 Primary analyses: associations among protective factors and children’s mental health

For our primary analyses (see Table 2), we entered child age along with the protective factors of child coping, parental resilience, and social supports as independent variables in our regression models and mental health outcomes as the dependent variables. There was a significant effect of child coping on pandemic-related distress, such that greater use of coping strategies related to lower distress (β = −0.25, *p* < 0.05); 95% CI [−21.2, 0.02]. Parental resilience had a significant effect on children’s mental health concerns, such that higher parental resilience was associated with lower likelihood of children’s mental health concerns worsening during the pandemic (β = −0.28, *p* < 0.05); 95% CI [−0.67, −0.06]. Finally, parental perceptions of social support had significant effects on several outcomes of interest, such that greater social support was associated with fewer mental health concerns (β = −0.30, *p* < 0.01); 95% CI [−0.42, −0.05], mental health emergencies (β = −0.25, *p* < 0.05); 95% CI [−0.21, −0.01], and lower pandemic-related distress (β = −0.25, *p* < 0.05); 95% CI [−12.7, −0.07].

**Table 2:** Protective factors at individual, parental, and community level.

	Child coping			Parental resilience			Social supports		
	T	β	SE	T	β	SE	T	β	SE
*Mental health*									
Child MH concerns	−0.74	−0.09	0.20	−2.37	−0.28*	0.15	−2.64	−0.30**	0.10
Child MH emergencies	−1.06	−0.13	0.10	−1.57	−0.18	0.08	−2.21	−0.25*	0.05
*Pandemic-related distress*	−1.99	−0.25*	5.33	−1.58	−0.20	4.69	−2.02	−0.25*	3.16
*Negative social impact*	−1.73	−0.22+	0.25	0.733	0.01	0.20	0.68	0.09	0.12

*N* = 81. + *p* < 0.10, *p < 0.05, **p < 0.01, ***p < 0.001. Models adjusted for patient age.

## 4 Discussion

The present study had two major aims—first, to analyze service usage within an outpatient child and adolescent psychiatry clinic during the COVID-19 pandemic, and second, to identify possible protective factors against mental health concerns in a sample of treatment-engaged youth. Findings suggest higher service usage during the COVID-19 pandemic and that children’s use of coping strategies, parental resilience, and parental perceptions of social connectedness were associated with children’s lower mental health concerns, emergencies, and/or pandemic-related distress.

That our findings demonstrated an uptick in the number of pediatric outpatient psychology and psychiatry appointments in the months during the COVID-19 pandemic is unsurprising, yet few studies have published this data within an ambulatory care setting. Most research has focused instead on the substantial increase in pediatric ED visits for mental health conditions throughout the course of the pandemic (e.g., Radhakrishnan, 2022). What is less clear from our study and extant research is the outpatient clinician’s role and relative success in managing higher caseloads and more complex patient presentations to prevent and reduce the number of ED visits —an area warranting further investigation. Additionally, the higher volume of patients in our outpatient clinic was largely accommodated by the expansion of telemedicine and emergency laws authorizing and reimbursing for remote practice (e.g., CARES Act). The increase in volume and utilization of telehealth services has remained even after the return to in-person activities and relaxing of pandemic-related restrictions (e.g., mask mandates; Lee et al., 2023). Research increasingly points to the benefits of telehealth in expanding coverage and optimizing care, with results comparable to conventional in-person therapies (Enebrink et al., 2012).

We also found associations between children’s use of coping strategies and lower pandemic-specific distress. This finding suggests that coping tools and strategies, many of which are learned or practiced within therapy, may be effective in managing some of the uncertainty and negative emotions associated with the pandemic. At the parental level, parents with higher self-reported psychological resilience were less likely to rate their children’s mental health as worsening. This result is consistent with a growing literature (e.g., Russell et al., 2021; Urban et al., 2022) that illustrates the impactful role of parents in facilitating children’s adjustment during the pandemic, lending support for the use of family-based interventions to promote both child and parent coping and self-efficacy.

At the community level, parental report of perceived social support was linked to children’s fewer mental health concerns and emergencies and lower distress. Social support has been well-established as a buffer for children and families against adverse events, including psychological distress associated with the COVID-19 pandemic (e.g., Kurudirek et al., 2022). Still, results suggest that even strong social supports may not wholly protect against children’s negative social impact due to the pandemic. Neither individual coping strategies, parental resilience, nor strong social supports could prevent the sense of loss experienced by youth—both in missing important milestone events and facing isolation from friends and family due to social distancing requirements.

Study findings must be evaluated in the context of its limitations. While the survey was disseminated widely in our clinic, we had a low response rate, small sample size spanning a large age range, and offered the survey in English only —all factors that limit the generalizability of the results. Further, our data included parent ratings only, including reports of parental perceptions of their own social support rather than ratings of their children’s social supports. Findings would have been strengthened by the addition of youth perspectives.

This study extends the COVID-19 literature by highlighting the increased service demands of an outpatient pediatric psychiatry clinic during the pandemic and demonstrating associations between protective factors at the individual child, parental, and community level and lower distress and mental health concerns and emergencies in a sample of treatment-engaged youth. The long-reaching physical, emotional, and social effects of the COVID-19 pandemic on all youth in the United States warrant ongoing investigation. For those children and teens with mental health vulnerabilities, the optimization of individual, parental, and community-based resources may go a long way in enhancing psychological functioning and overall quality of life.

## Data availability statement

The datasets presented in this article are not readily available because of the Hospital’s Institutional Review Board’s policies. Requests to access the datasets should be directed to ajcaruso@mgh.harvard.edu.

## Ethics statement

The studies involving humans were approved by Massachusetts General Hospital Institutional Review Board. The studies were conducted in accordance with the local legislation and institutional requirements. Written informed consent for participation in this study was provided by the participants’ legal guardians/next of kin.

## Author contributions

AC: Writing – review & editing, Writing – original draft, Formal analysis, Conceptualization. AB: Writing – review & editing, Validation, Supervision, Methodology, Investigation, Formal analysis, Conceptualization. TU: Writing – review & editing, Methodology, Investigation, Conceptualization. MK: Writing – review & editing, Project administration, Methodology, Investigation, Formal analysis, Conceptualization. NR: Writing – review & editing, Project administration, Investigation, Conceptualization. JW: Writing – review & editing, Investigation, Funding acquisition, Conceptualization. DF: Writing – original draft, Methodology, Investigation, Formal analysis, Data curation, Conceptualization.
